# Supplementary material for: Rapid turnover of life-cycle-related genes in the brown algae
Source: Genome Biol. 2019 Feb 14;20:35. doi: 10.1186/s13059-019-1630-6 (PMC6374913; doi:10.1186/s13059-019-1630-6)
Supplement: Supplementary file 3 — Protein alignments, corresponding Gblocks html file and CDS sequences for the lineage conserved gametophyte-biased genes. (ZIP 2109 kb) [file 13059_2019_1630_MOESM3_ESM.zip › Con_GA_alignments/Ec-23_000390.aa_ali.fasta-gb.htm]

Ec-23\_000390.aa.fasta.trans.aa\_ali.fasta


## Gblocks 0.91b Results

Processed file: **Ec-23\_000390.aa.fasta.trans.aa\_ali.fasta**  
Number of sequences: **2**  
Alignment assumed to be: **Protein**  
New number of positions: **326** (selected positions are underlined in blue)

```
                         10        20        30        40        50        60
                 =========+=========+=========+=========+=========+=========+
Ec-23_000390     MDTRSSLWHSWPAKAEEEENGNGSRSTHQRQRQDVQQDAAATAATTGGTAVLRGTKYTSL
MSTRG.38049.1    ------------------KG--------------GNEHWDGPGVTDGKT-----------
                                                                             


                         70        80        90       100       110       120
                 =========+=========+=========+=========+=========+=========+
Ec-23_000390     QVALNMHASGVRFASGSVNSISAQECGLVSGDKLQRGGGILHVDSGRKHQRQHSLPRFVA
MSTRG.38049.1    ------------------------------------------------------------
                                                                             


                        130       140       150       160       170       180
                 =========+=========+=========+=========+=========+=========+
Ec-23_000390     KVFLVSSAIVVAAMLVIAHQRKGWSGSGAVLPSHGNDRATATATALVQRGNSKGRFRSEG
MSTRG.38049.1    ----------------------------------MTIKGLKGAGVAALAPHSRGIPRSEG
                                                           ##################


                        190       200       210       220       230       240
                 =========+=========+=========+=========+=========+=========+
Ec-23_000390     LHDQNDREHLDAASIPASSRAPS-SVSRALGDMPITTAPDPGGDGDGGGGAAT-TYEKRA
MSTRG.38049.1    LHSLNDNEPLAEALLSSPAMHRSRYLGIPEKSTAASTASAVGGSGGSGSAKSTNTLEKRA
                 #############                       ################# ######


                        250       260       270       280       290       300
                 =========+=========+=========+=========+=========+=========+
Ec-23_000390     RIGYLIMSSGTEELHKTKRLLKAIYDPNNFYLVHLDRKDKRSIRRDFENFIEEWDNVRML
MSTRG.38049.1    RIGYLIMASGVEELHKTKRLLQAIYDPENFYLVHLDRKDNRAIRRDIENFIKDWDNVRML
                 ############################################################


                        310       320       330       340       350       360
                 =========+=========+=========+=========+=========+=========+
Ec-23_000390     EPALDVSWGGYTITLTAIFGICTMVQWNDEWDFFINLSASDFPLLPQSELTTVLGKYADV
MSTRG.38049.1    VPSLDVSWGGYTVTLTAIFGICTLVQWSNEWDYFINLSASDFPLLPQSELTTVLGQYAGM
                 ############################################################


                        370       380       390       400       410       420
                 =========+=========+=========+=========+=========+=========+
Ec-23_000390     GMNFVSGEPLNERNRVEVLIDDQGLYREKQSSKAGRPLKVGKARLPPSKSMFTVYKGEFW
MSTRG.38049.1    GMNFVSGEPLNERNRVEVIIDDQGLYRETQSSKAGRPLKVGKPRLPPSKSMFTVYKGEFW
                 ############################################################


                        430       440       450       460       470       480
                 =========+=========+=========+=========+=========+=========+
Ec-23_000390     VILHRSFCQYLEASPDNVARSLQAYFSKFRISDESYFQTVLCHPLAPSFLVHPDNLRFVS
MSTRG.38049.1    VILHRSFCQYIEKSPDNVARSLQAYFSRFRISDESYFQTVLCHPLAPSFRVHPDNLRFVS
                 ############################################################


                        490       500       510       520       530       540
                 =========+=========+=========+=========+=========+=========+
Ec-23_000390     WPDVIEGHYVLHPDPITGGASGNVNVAMDSGALFARKFDTKVSQEAYTVLEKSLSEPNPG
MSTRG.38049.1    WPLVSEGHYVLHPDPIMGGTNGNVNVAMESGA----------------------------
                 ################################                            


                        550       560       570       580       590       600
                 =========+=========+=========+=========+=========+=========+
Ec-23_000390     RLARAANRLNPAAAVTGRQRFCFVPDGYSIQEVLSRPPKKAMGMHQDRSDGSSSNRNRDG
MSTRG.38049.1    ------------------------------------------------------------
                                                                             


                        610       620       630
                 =========+=========+=========+==
Ec-23_000390     SSSPLAVGKDQLQRQPSFQTREAREGEGGSEP
MSTRG.38049.1    --------------------------------
```

```
Parameters used
Minimum Number Of Sequences For A Conserved Position: 2
Minimum Number Of Sequences For A Flanking Position: 2
Maximum Number Of Contiguous Nonconserved Positions: 8
Minimum Length Of A Block: 10
Allowed Gap Positions: None
Use Similarity Matrices: Yes
```

```
Flank positions of the 3 selected block(s)
Flanks: [163  193]  [217  233]  [235  512]  

New number of positions in Ec-23_000390.aa.fasta.trans.aa_ali.fasta-gb:  326  (51% of the original 632 positions)
```
